# Improvement of mechanical, humidity resistance and thermal properties of heat-treated rubber wood by impregnation of SiO_2_ precursor

**DOI:** 10.1038/s41598-018-37363-3

**Published:** 2019-01-30

**Authors:** Nannan Zhang, Min Xu, Liping Cai

**Affiliations:** 1 Key Laboratory of Bio-based Material Science & Technology (Northeast Forestry University), Ministry of Education, Harbin, 150040 P.R. China; 2 Mechanical and Energy Engineering Department, University of North Texas, Denton, Texas 76201 USA; 3 Nanjing Forestry University, Nanjing Forestry University, Nanjing, Jiangsu 210037 China

## Abstract

The SiO_2_ precursor solution was impregnated into heat-treated rubber wood to enhance its mechanical and flame-retarding properties. Test specimens were randomly divided into four groups, i.e., untreated (U), heat-treated (HT), impregnated SiO_2_ precursor before heat treatment (ISB) and after heat treatment (ISA). Results showed that, compared with HT wood specimens, the modulus of rupture (MOR) and compression strength of ISB and ISA wood specimens were both increased. The hygroscopicity of modified wood was decreased and the dimension stability was consequently improved. Surprisingly, the hardness of ISB specimens increased by 43.65%. The thermogravimetric (TG) examination showed that the incorporation of silicon retarded the thermal decomposition and improved the thermal stability of wood. Furthermore, the scanning electron microscopy (SEM) and energy dispersive X-ray analysis (EDXA) revealed that the SiO_2_ gel was deposited in the cell wall, The Fourier transform infrared spectroscopy (FTIR) showed the formation of Si–O–Si and Si–O–C covalent bonds. The X-ray diffraction (XRD) tests indicated that the impregnation of SiO_2_ precursor had slight effect on the crystalline structure of the wood.

## Introduction

As a renewable biomaterial, wood has been widely used in construction, furniture and packaging owing to its workability and sustainability. However, there are still many problems to limit its applications. For examples, owing to its inherently hygroscopic and organic constitution, wood is susceptible to humidity change and low fire-retardancy, resulting in dimensional and thermal instability^1–3^. As a nutrient source, wood is vulnerable to insects, fungi and other microorganisms^4,5^. To address these drawbacks, various techniques have been developed to modify wood, such as acetylation^6^, furfurylation^7^, DMDHEU^8^ and heat treatment. While these methods have their own advantages, only a few have found industrial applications^9^. There are still downsides limiting applications, such as low effectivity, high costs and poor mechanical properties and negative environmental impacts^10^.

Heat treatment (HT) is an eco-friendly modification process, which can decrease wood wettability^11,12^, and improve dimensional stability^13^ and durability^14^. The chemical composition and structure of wood were changed in HT, resulting in a severe loss in mass and mechanical properties^15–18^. Many technologies have been proposed to alleviate the negative effects of heat treatment, such as combining the heat treatment with the treatment of boron^19,20^, wax emulsion^21^, zinc oxide nanoparticles^22,23^ or nitrogen-phosphorus fire retardant^24^.

Sol–gel derived wood-inorganic composite is a promising material due to its enhanced properties and environmental-friendly application^25,26^. Over the last several years, many inorganic compounds have been applied for wood modification, such as SiO_2_ and TiO_2_^27,28^. SiO_2_-wood composites exhibited a flame retardancy, dimensional and UV stabilization as well as antimicrobial properties^29–33^. These improvements are due to the stable incorporation of the inorganic components in the wood substrate. Although the inorganic nanosols have been used to surface finishing of heat-treated wood^34^, the application for improving mechanical properties of the heat-treated wood has not been discovered in the literature review.

This study proposed a novel methodology by impregnating SiO_2_ precursor solution into the heat-treated wood to improve the mechanical properties. Subsequent the synergy of heat treatment and silica gel on hygroscopicity as well as dimensional and thermal stability were also investigated. The SEM/EDAX examinations, and FTIR and XRD analyses were used to examine the effect of the combination of silica and wood on the panel properties.

## Results and Discussions

Table 1 shows that, the data of four groups were normally distributed (Shapiro-Wilk test: p > 0.05, two-sided) at the significance level of 0.05, so the independent samples t test (two-tailed) was used to analyze the difference in mechanical properties between different treatments at the significance level of 0.05 (Table 2). The results indicated that, compared with the untreated wood specimens, the compression strength, modulus of rupture (MOR), modulus of elasticity (MOE) of heat-treated (HT) specimens were decreased by 18.21%, 48.95%, and 9.38%, respectively. It was found that the compression strength (P = 0.018) and MOR (P = 4.74 × 10^−6^) of rubber wood were significantly affected by HT, while the effects on the MOE (P = 0.254) were insignificant. It was indicated that the heat treatment could decrease the mechanical properties of wood, which was consistent to the previous studies^13^. It was mainly due to the degradation of hemicellulose, which degraded firstly in the heat treatment process because of the low degree of polymerization and the amorphous structures^35,36^. Compared with the heat-treated specimens, the compression strengths of impregnated SiO_2_ precursor after heat treatment (ISA) and before heat treatment (ISB) specimens were insignificantly increased (P = 0.084) by 17.96%, while the ISB were significantly increased (P = 0.003) by 33.64%. The MOR of ISA and ISB specimens were significantly increased (P = 4.86 × 10^−5^) by 43.87%, the ISB specimens were insignificantly increased (P = 0.088) by 20.37%, while the MOE of ISA (P = 0.733) and ISB (P = 0.704) specimens were insignificantly increased. Compared with the untreated wood specimens, the hardness of HT specimens was insignificantly increased (P = 0.365). Compared with heat-treated specimens, the and ISB specimens significantly increased (P = 6.7 × 10^−5^) by 43.65%, this was improved by incorporating the SiO_2_ gel into cell walls, which delayed the heat transfer and reduced the degradation and destruction of the heat-treated wood as well as improved the hardness of cell walls. While the hardness of ISA specimens significantly (P = 0.021) decreased. This was due to the stabilizing compounds in the wood were dissolved by the ethanol-containing solvent sol^4^.

**Table 1:** Mechanical properties and the Shapiro-Wilk test results of wood specimens with different treatments (n = 7).

Treatments	Compression strength (MPa)	p	MOR (MPa)	p	MOE (MPa)	p	Hardness (N)	p
U	66.54 ± 5.19	0.189	102.63 ± 7.91	0.705	7202.83 ± 817.03	0.248	3662.52 ± 167.62	0.789
HT	54.42 ± 9.46	0.069	52.39 ± 4.36	0.869	6527.54 ± 1112.79	0.095	3788.95 ± 309.15	0.447
ISA	64.19 ± 8.58	0.536	75.37 ± 3.88	0.580	6743.61 ± 693.26	0.616	3454.78 ± 216.51	0.519
ISB	72.73 ± 1.79	0.945	64.56 ± 9.56	0.152	6792.79 ± 1244.39	0.649	5261.28 ± 411.79	0.983

**Table 2:** Comparison effects of different treatment using t test (two-tailed) on the mechanical properties (n = 7).

Sources	Compression strength		MOR		MOE		Hardness	
	t	P	t	P	t	P	t	P
U-HT	2.751	0.018	11.75	0.000	1.198	0.254	−0.936	0.365
HT-ISA	−1.920	0.084	−7.881	0.000	−0.351	0.733	2.571	0.021
HT-ISB	−4.659	0.003	−2.067	0.088	−0.389	0.704	−8.427	0.000

**Figure 1:**
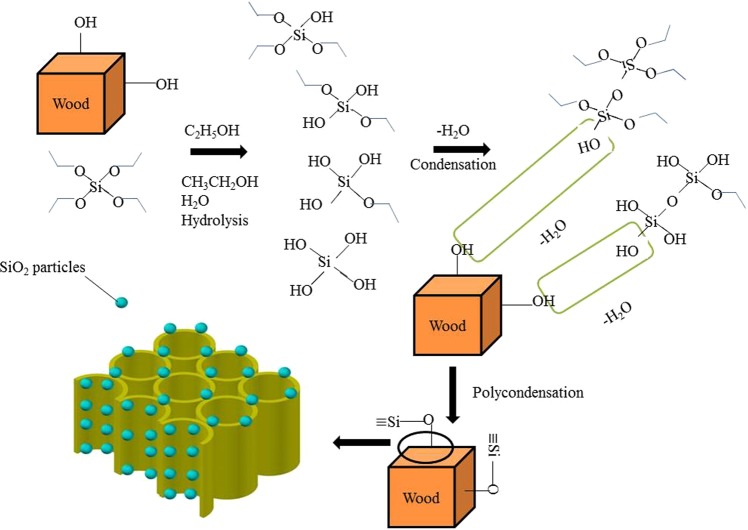
Schematic illustration of the preparation of SiO_2_ modified wood.

The results indicated that the impregnation of SiO_2_ precursor could compensate the mechanical loss caused by the heat treatment. The precursor was immersed into the wood and TEOS hydrolyzed utilizing OH groups of wood substrate and condensed to SiO_2_ gel during the heat treatment process as shown in Fig. 1. The SiO_2_ was adhered to the wood matrix and crosslinked with each other, which delayed the heat transfer and reduced the degradation and destruction of the heat-treated wood. The SiO_2_ gels were deposited in wood matrix as fillers, which densified and stiffened the cell walls, and strengthened the resistance of the cell wall against from the deformation and destruction. At the same time, the hydrogen bonds between the SiO_2_ and wood hydroxyl groups, as well as the crosslinking also provided compensation for the mechanical loss.

The dry mass loss by HT treatment was 5.44%, which attributed to the hemicellulose decomposition and volatilization of extraction. The dry mass gains by SiO_2_ impregnation before and after heat treatment were 3.62% and 11.03% respectively. The dry mass gain of ISB was less than ISA, probably because the internal porosity of the wood increased after the heat treatment, which provided more space for silica impregnation. The oven-dry dimension gains by the ISA and ISB treatments were 1.07% and 2.16% respectively, illustrating the bulking of cell walls.

**Figure 2:**
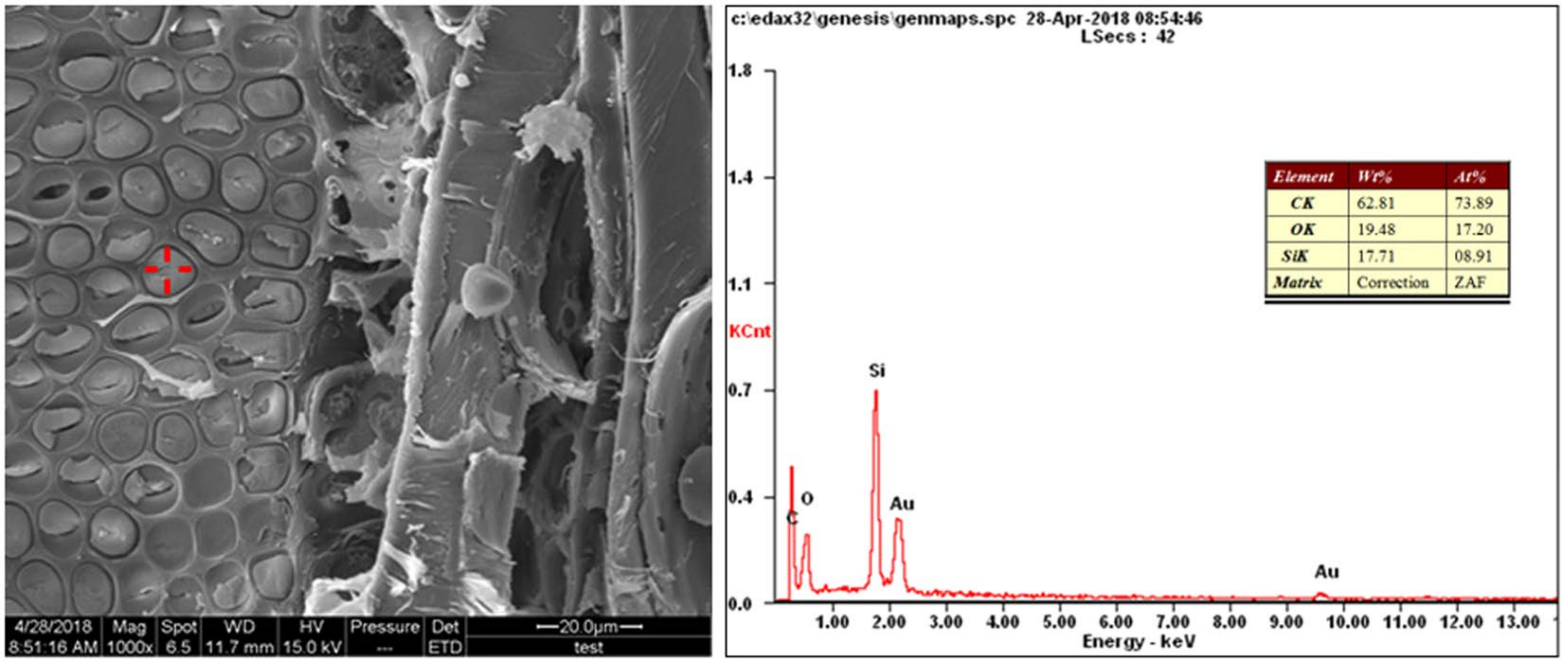
SEM and EDXA spectra of the ISA wood specimen.

The composition of the ISA wood surface was analyzed using the energy dispersive X-ray analysis (EDXA) spectra and the responding scanning electron microscopy (SEM) images, and are presented in Fig. 2. The pits in the vessel of the modified wood were covered by silica gel. The appearance of a strong peak at approximately 1.8 keV indicated the presence of silicon on the surface. In other words, the TEOS precursor could penetrate the cell wall before hydrolysis and polycondensation^37^, suggesting that the SiO_2_ gel formed and deposited in the cell walls. The Si content was 17.71 wt. %.

**Figure 3:**
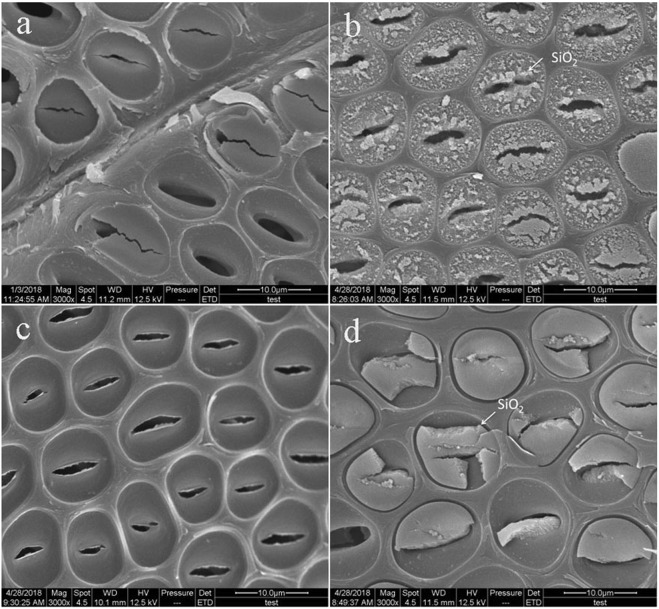
SEM images of (**a**) the untreated, (**b**) ISB, (**c**) HT and (**d**) ISA wood specimens.

Figure 3 shows the surface morphologies of wood specimens. In Fig. 3(a), the untreated wood exhibits a smooth surface with some clearly visible pits, and some pits have a pit membrane. Figure 3(c) presents the surface morphology of the heat-treated specimens, which also have some visible pits, illustrating that no significant change occurred compared with that of the control ones. In Fig. 3(b), the pits were covered by nanoscale particulates in ISA wood surface. Figure 3(d) shows the pits were filled with packed gels.

**Figure 4:**
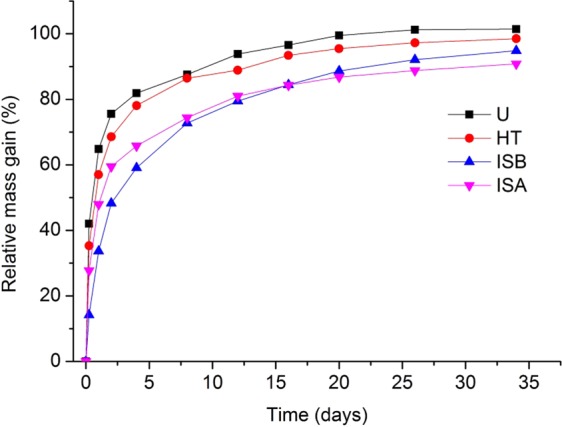
Hygroscopicity curves of wood specimens with different treatments.

As shown in Fig. 4, the moisture uptake of all wood specimens increased with time. All the wood specimens absorbed water quickly within the first 10 days when the specimens were immersed in water, and the masses remained almost constant after a month immersion. During the period immersing in water, the gains in mass of the untreated specimens corresponded to a saturation of approximately 101% in 35 days. The moisture absorption of the HT, ISB, ISA specimens were lower than that of the untreated ones, in which, the ISB and ISA specimens were much lower, counting for reductions of 91%, 95%, respectively. It was indicated that the heat treatment can weaken the hygroscopicity of wood relatively and the impregnated SiO_2_ precursor with heat treatment further weakened the hygroscopicity. It was due to the degradation of hemicellulose and loss of other hydroxyl-containing components during the heat treatment, resulting in the reduction in the number of hydroxyl groups^2^. On the other hand, a part of the hydroxyl groups possibly was blocked by the formation of hydrogen bonds with SiO_2_ gels during the hydrolysis and polycondensation of TEOS precursor, thereby weakening the hygroscopicity. Additionally, SiO_2_ could amass in the cell wall pores as fillers, as shown in Fig. 3(b,d), which hindered the moisture absorption. Therefore, the moisture absorption of ISB and ISA were reduced.

**Figure 5:**
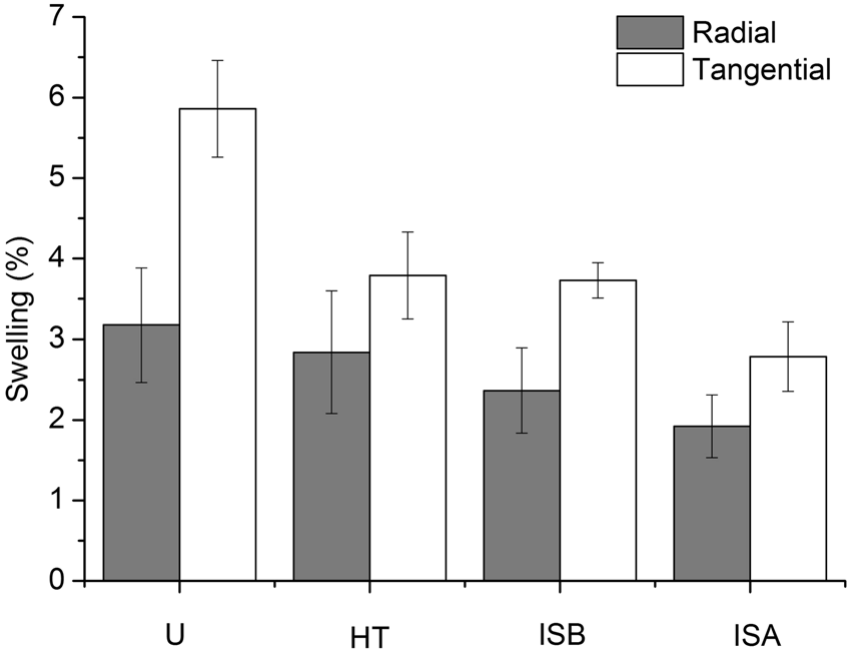
Swelling of wood specimens with different treatments.

Figure 5 shows the swelling of wood specimens with different treatments. Compared with the untreated wood specimens, the radial swelling rates of the HT, ISB and ISA specimens were decreased by 10.53%, 25.47% and 39.47%, respectively, and the tangential swelling rates were decreased by 35.33%, 36.37% and 52.48%, respectively. The swelling of the ISA and ISB specimens were lower than that of HT specimens. It was indicated that the heat treatment could improve the dimensional stability, and the incorporation of SiO_2_ gels helped to reduce the swell ability of cell walls, resulting in the improvement of dimensional stability. The ISA samples swelled less than the ISB samples, which was attributed to the amount of SiO_2_ impregnation. The dry mass gains by SiO_2_ impregnation of ISA and ISB samples were 11.03% and 3.62%, respectively. The more amount of SiO_2_ impregnation of ISA caused the lower amount of accessible OH groups, which helped to reduce the swell ability of cell walls.

**Figure 6:**
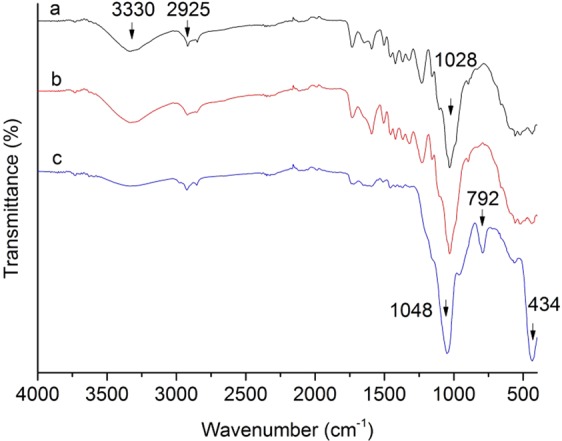
FTIR spectra of (**a**) untreated, (**b**) HT and (**c**) ISB wood specimens.

Figure 6 shows the Fourier transform infrared spectroscopy (FTIR) spectra of three types of wood specimens, namely, the untreated samples, the HT samples, and the samples with impregnation of SiO_2_ precursor before HT. In Fig. 6(a), the untreated rubber wood absorption peaks mainly include: 3330 cm^−1^ (-O-H stretching vibration), 2925 cm^−1^ (-C-H stretching vibration) and 1028 cm^−1^ (-C-O stretching vibration). The peak intensities of ISB and HT at 3330 cm^−1^ (-O-H stretching vibration) were significant lower as shown in Fig. 6(b,c), It was because that the heat treatment led to the reduction of hydroxyl groups, and the wood–OH reaction groups might combine with silica nanoparticles to form Si–O–Si or Si–O–C covalent bonds. The Si–O–Si bonds provided three characteristic FTIR absorption bands in Fig. 6(c): 434 cm^−1^, 792 cm^−1^, 1048 cm^−1^, and the Si-O-Si symmetric stretching vibration absorption peak appeared at 1048 cm^−1^ overlapped with Si-O-C bonds^38^. The intensity of band at 1731 cm^−1^ reduced due to the degradation of hemicellulose[3], and the disappearance of the signature at 1239 cm^−1^ could be because of the overlap with the Si-O-C bonds.

**Figure 7:**
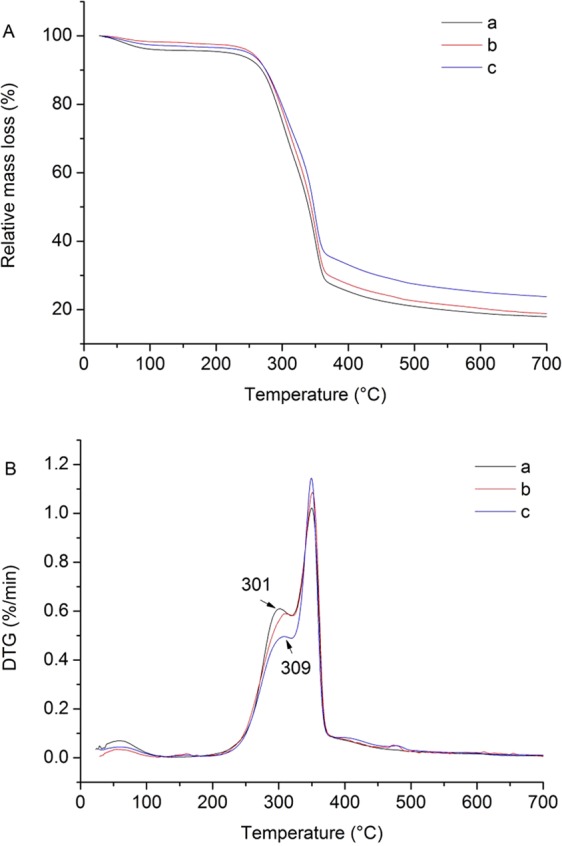
Thermal behavior (TG curves and DTG curves) of (**A**) untreated, (**B**) HT and (**C**) ISB wood specimen.

In Fig. 7(A), thermogravimetric (TG) curves showed that the thermal behavior of rubber wood was divided into four stages, in which, the initial weight loss was mainly attributed to the evaporation of wood moisture from room temperature to 200 °C. An abrupt weight loss was observed between 200–320 °C, followed by the another obvious weight loss between 320–375 °C. It was due to the degradation of hemicellulose followed by cellulose and lignin; the residual wood components continued to aromatize and carbonize above 400 °C. The amount of the final residue of the U, HT and ISB specimens were 17.27%, 18.29%, and 23.07%, respectively, indicating that the proportion of the solid char obtained by the heat treatment increased, and there was a residue such as silica that had not yet been decomposed at 700 °C.

There are two characteristic peaks at the derivative thermogravimetric (DTG) curves in Fig. 7(B). The first peak of the U specimens located at 301 °C, while the HT and ISB specimens were at 309 °C. The rate of weight change of ISB specimens was lower than that of HT specimens, and the second peak were all around at 349 °C. The first peaks of ISB and HT specimens were shifted to a higher temperature, indicating that the heat treatment and impregnation of SiO_2_ precursor could improve the thermal stability of wood. The first peak of ISB was weakened in comparison with the HT specimens, which was because the silica gels adhered to the cell walls of the wood and acted as a barrier to oxygen and retarded their combustion.

**Figure 8:**
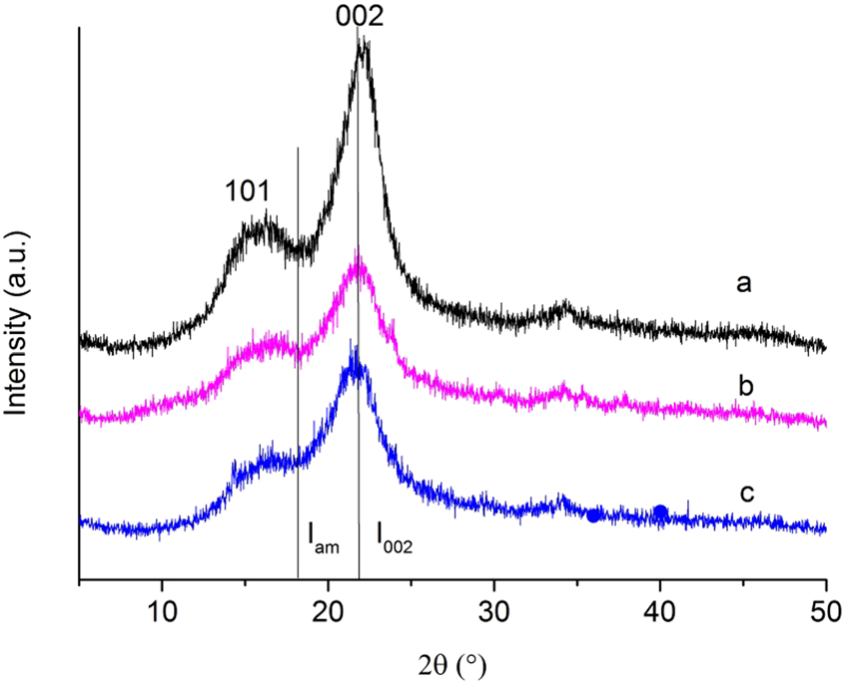
XRD patterns of (**a**) the untreated, (**b**) the HT and (**c**) the ISB wood specimens.

The characteristic diffraction peak of cellulose (2θ = 15, and 22°) appears in Fig. 8(a). It was shown that the relative crystallinity of the untreated, HT and ISB specimens were 73.5%, 59.47% and 61.46%, respectively. The relative crystallinity of the heat-treated wood decreased, because various acids such as acetic acid formed due to the hydrolysis of hemicellulose at 200 °C. These acids acted as catalyses in the degradation of amorphous of cellulose even in the crystalline area, which reduced the relative crystallinity of wood. The relative crystallinity of ISB specimens were higher than that of HT specimens, indicating that the impregnated SiO_2_ precursor did not destroy or even change the crystalline structure of cellulose.

## Conclusions

The effects of SiO_2_ on the mechanical properties and hygroscopicity, as well as the thermal stability of the heat-treated rubber wood were investigated. The conclusions were drawn as follows.

1. After the heat treatment, the wood mechanical properties decreased due to the degradation of substrates. Impregnation of SiO_2_ precursor before heat treatment delayed the heat transfer and reduced the degradation, and hence compensated the loss of the mechanical properties caused by heat treatment. The incorporation of SiO_2_ improved the MOR and compression strength of the heat-treated wood.
2. The hygroscopicity of SiO_2_-modified heat-treated wood was decreased, and the dimension stability was improved because the SiO_2_ amassment in the cell walls blocked the moisture absorption.
3. The incorporation of silica and wood retarded the thermal decomposition, resulting in the improved thermal stability.

Therefore, the application of silica nanosols on heat-treated wood could lead to improved mechanical properties and thermal stability, as well as dimensional stability. This may be further considered as an efficient method to extend the utilization of heat-treated wood.

## Materials and Methods

### Materials

Seven wood specimens with the longitudinal grain direction for each group were obtained from the sapwood of rubber wood (*Hevea brasiliensis*) at a local sawmill in Hainan Province, China. Tetraethyl Orthosilicate (C_8_H_20_O_4_Si ≥ 97.09%) was purchased from Tianjin Fuchen Chemical Reagents Factory, Tianjin, China. Acetic acid (CH_3_COOH, ≥99.5%) was purchased from Tianjin Tianli Chemical Reagents Co., Ltd. Ethanol (C_2_H_5_OH, ≥99.7%) was purchased from Tianjin Fuyu Chemical Co., Ltd. All chemicals were analytical grades. Wood specimens were randomly divided into four groups, i.e., untreated (U), heat-treated (HT), impregnated SiO_2_ precursor before heat treatment (ISB), and impregnated SiO_2_ precursor after heat treatment (ISA) specimens.

The SiO_2_ precursor solutions were produced according to the following procedure. The TEOS was dissolved in the ethanol and stirred at room temperature for 10 min, then the deionized water and acetic acid as catalysts were added in the solution with a molar ratio of n (H_2_O): (TEOS): n (C_2_H_5_OH): n (CH_3_COOH) = 1:1:1:0.01.

### Impregnation and heat treatment

The wood specimens were impregnated with precursor solution at a vacuum of 0.08 MPa absolute pressure for 2 h. The heat treatment was performed at 200 °C for 2 h with a temperature-controlled laboratory oven.

### Physical and mechanical properties test

The universal mechanical test machine (Changchun Kexin Instrument Co., Ltd. AG-10TA) was used for mechanical property tests. The specimens with a size of 20 mm × 20 mm × 30 mm were utilized for measuring the compression strength with a crosshead loading speed of 2 mm/min. The specimens with a size of 20 mm × 20 mm × 300 mm were used for measurements of MOR and MOE, in which, three-point bending was set-up with a span of 240 mm and a crosshead speed of 5 mm/min., The specimens of 50 mm × 50 mm × 70 mm were used for measuring the hardness, with a crosshead speed of 4 mm/min. Seven replicates were completed for each group of specimens.

To evaluate the moisture absorption and swelling property, five specimens for each group were dried at 60 °C until the constant masses were reached, and the masses and the dimensions of radial (R), tangential (T) and longitudinal (L) were measured. Then the specimens were submerged in glass containers with deionized water at 20 °C, and the relative mass gains were calculated based on the weight differences between the specimens before and after the moisture absorption period divided by the initial specimen values. After the stable dimensions were obtained in 20 days, the dimensions were measured again. The swelling rates of radial(R), tangential (T) and longitudinal (L) were based on the differences between the specimens before and after the moisture absorption divided by the initial specimen values, respectively. The oven-dry dimension gains by the ISA and ISB treatments were based on the differences between the specimens after SiO_2_ the impregnation divided by the initial specimen values, respectively.

### Characterization

The Fourier transform infrared spectroscopy (FTIR, Termo Fisher Scientifc, and Nicolet 6700) measurements were used to explore the changes of chemical composition. The wood specimens were examined in the range of 4000–400 cm^−1^ with a resolution of 4 cm^−1^ and 32 times of scans for each spectrum. The crystalline structure was analyzed by the X-ray diffraction (XRD, Philips, and D/max2200) operating with Cu radiation and at the acceleration voltage of 40 kV, the current of 30 mA, the scanning range (2θ) from 5 to 50°, and the scan rate of 4°/min. The thermal properties of wood specimens were examined by a thermal analyzer (TGA, Q50) in the temperature range from room temperature to 700 °C at a heating rate of 10 °C/min.

Specimens were sputter-coated with gold layer, and the morphology of wood sample surface was characterized by the scanning electron microscopy (SEM, FEI and Quanta200). The chemical composition of the wood surface was determined using the energy dispersive X-ray analysis (EDAX, FEI, and Quanta200) connected with the SEM.

### Statistical analyses

Shapiro-Wilk test (two-sided) was used to identify the normality of the data at the 0.05 significance level. The t tests (two-tailed) were performed to analyze the difference in the mechanical properties between untreated and heat-treated rubber wood and the effects of impregnation SiO_2_ precursor treatment on the heat-treated rubber wood at the 0.05 significance level.

## Data Availability

All data generated or analyzed during this study are included in this published article.
